# Supplementary material for: A yeast phenomic model for the influence of Warburg metabolism on genetic buffering of doxorubicin
Source: Cancer Metab. 2019 Oct 23;7:9. doi: 10.1186/s40170-019-0201-3 (PMC6806529; doi:10.1186/s40170-019-0201-3)
Supplement: Supplementary file 9 — Additional file 9. GO term-specific heatmaps for REMc/GTF-enriched clusters. GO term-specific heatmaps for significant GO process terms were generated as described in methods and Figs. 3 and 4. Any related child terms are presented in subsequent pages of the parent file name. GO terms with more than 100 children, with 2 or fewer genes annotated to the term, or a file size over 300KB are not shown. All heatmaps are generated with the same layout (see Figs. 3 and 4). [file 40170_2019_201_MOESM9_ESM.bz2 › Additional_File9_GOTermHeatmaps/Additional_File9_GOTermHeatmap/positive_regulation_of_phospholipid_metabolic_process.pdf]

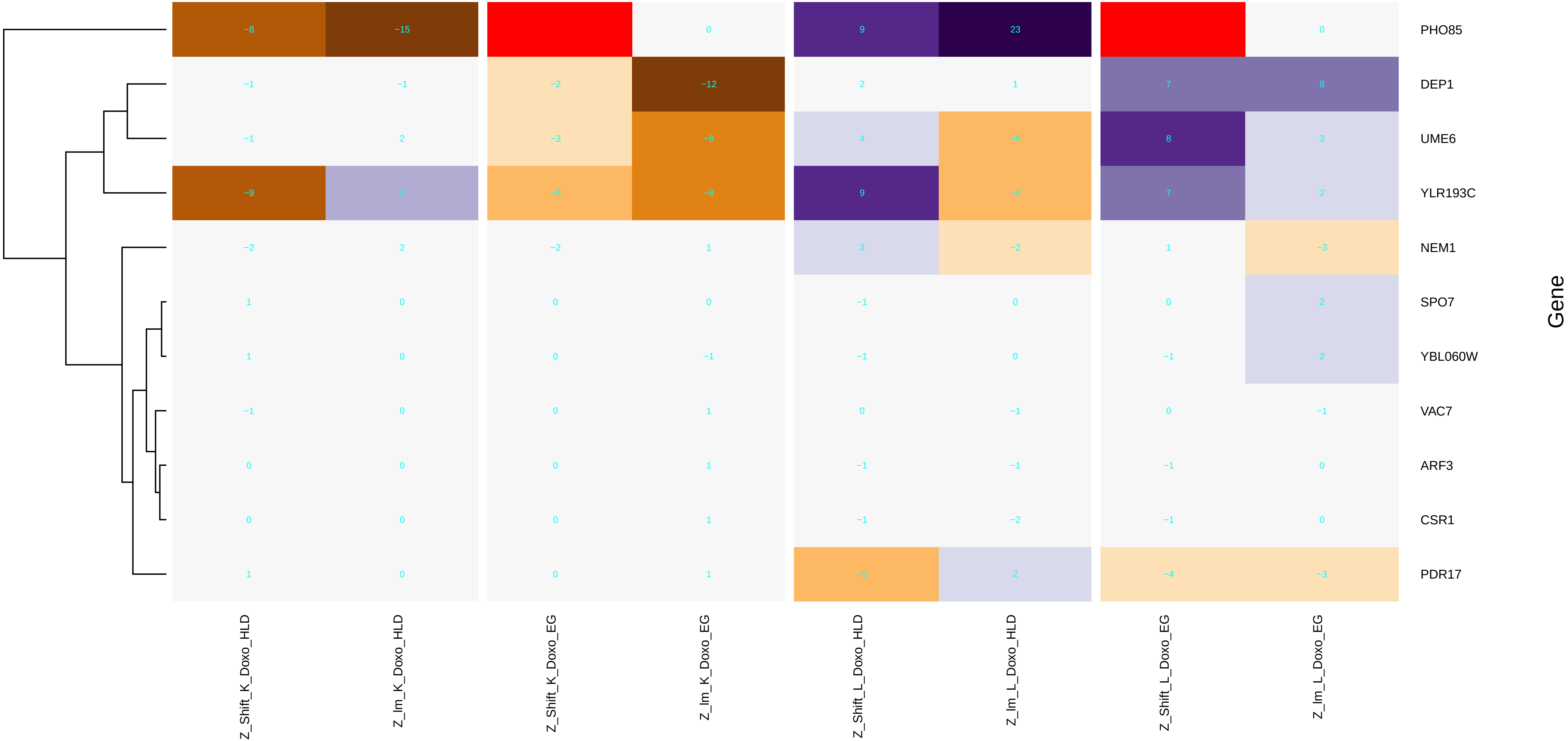
PHO85
-8 -15 0 9 23 0
DEP1
-1 -1 -2 -12 2 1 7 8
UME6
-1 2 -3 -8 4 -5 8 3
YLR193C
-9 4 -5 -8 9 -4 7 2
NEM1
-2 2 -2 1 3 -2 1 -3
SPO7
1 0 0 0 -1 0 0 2
YBL060W
1 0 0 -1 -1 0 -1 2
VAC7
-1 0 0 1 0 -1 0 -1
ARF3
0 0 0 1 -1 -1 -1 0
CSR1
0 0 0 1 -1 -2 -1 0
PDR17
1 0 0 1 -5 2 -4 -3
Gene
Z_Shift_K_Doxo_HLD
Z_Im_K_Doxo_HLD
Z_Shift_K_Doxo_EG
Z_Im_K_Doxo_EG
Z_Shift_L_Doxo_HLD
Z_Im_L_Doxo_HLD
Z_Shift_L_Doxo_EG
Z_Im_L_Doxo_EG
